# Microencapsulation of Dandelion (*Taraxacum officinale* L.) Leaf Extract by Spray Drying

**DOI:** 10.17113/ftb.60.02.22.7384

**Published:** 2022-06

**Authors:** Arijana Martinić, Ana Kalušević, Steva Lević, Viktor Nedović, Aleksandra Vojvodić Cebin, Sven Karlović, Igor Špoljarić, Gordan Mršić, Krunoslav Žižek, Draženka Komes

**Affiliations:** 1 University of Zagreb, Faculty of Food Technology and Biotechnology, Pierottijeva 6, 10000 Zagreb, Croatia; 2 University of Belgrade, Faculty of Agriculture, Nemanjina 6, 11080 Belgrade, Serbia; 3 Academy of Applied Studies Belgrade, Bl. Zorana Đinđića 152a, 11070 Belgrade, Serbia; 4 Forensic Science Centre ‘‘Ivan Vučetić’’ Zagreb and Forensic Science Office of University of Zagreb, Ilica 335, 10000 Zagreb, Croatia; 5 University of Zagreb, Faculty of Chemical Engineering and Technology, Marulićev trg 19, 10000 Zagreb, Croatia

**Keywords:** carriers, dandelion, encapsulation efficiency, physicochemical properties, polyphenols, spray drying

## Abstract

**Research background:**

Due to numerous health-promoting properties, dandelion has been used in traditional medicine as a herbal remedy, but also as a food product. Dandelion health benefits are ascribed to the presence of different bioactive compounds in its tissues, among which polyphenols play a significant role. However, the low stability of polyphenols is a critical parameter for their successful implementation into products. Thus, their encapsulation using appropriate carrier vehicles is highlighted as an effective technique for their stabilization and protection. The aim of this study is to microencapsulate dandelion leaf extract using spray drying and different carrier materials for the first time.

**Experimental approach:**

In spray drying, low inlet temperature of 130 °C was employed to preserve sensitive dandelion polyphenols, while guar gum, gum arabic, inulin, maltodextrin, pectin and alginate were used as carriers. The influence of different carriers and their content on physicochemical, morphological and colour properties, polyphenolic content and encapsulation efficiency of polyphenols in dandelion powders was examined. Specific polyphenols were determined using HPLC-PAD analysis. Their release profiles and antioxidant capacity in simulated gastrointestinal conditions were also evaluated.

**Results and conclusions:**

Compared to plain dandelion powder, carrier-containing dandelion powders have favourably increased solubility, enhanced flow and cohesive properties, reduced particle size and prolonged release of polyphenols under simulated gastrointestinal conditions. Powders were characterized by low moisture content (~2-8%) and high solubility (~92-97%). Chicoric acid was the most abundant compound in dandelion powders. Pectin-dandelion powder showed to be the most effective for microencapsulation of polyphenols, especially for chicoric acid entrapment (74.4%). Alginate-dandelion powder enabled the slowest gradual release of polyphenols.

**Novelty and scientific contribution:**

Spray drying at 130 °C and the applied carriers proved to be effective for microencapsulation of dandelion extract, where polyphenolic-rich dandelion powders, due to good physicochemical and encapsulation properties, could serve for the enrichment/production of different functional food products. Also, due to the lack of data on dandelion encapsulation, the obtained results could be of great interest for researchers in the encapsulation field, but also for food industry, especially in the field of instant powders.

## INTRODUCTION

Dandelion (*Taraxacum officinale* L. Weber ex F.H. Wigg), a nontoxic herb from Asteraceae family, has for centuries been used in a traditional medicine worldwide, mainly due to its antirheumatic, anti-inflammatory, anticarcinogenic, hepatoprotective, antioxidant and hypoglycaemic properties. These properties have been attributed to the large number of bioactive compounds found in dandelion tissues, like terpenes, flavonoids and phenolic compounds (*1*). Despite numerous benefits for human health, studies dealing with dandelion phytochemicals, especially polyphenols, are still limited. According to our search of the Web of Science databases, in the last few years only 25 scientific articles have studied polyphenols in dandelion (*Taraxacum officinale* L.) plant. In general, most of the studies are focused on the evaluation of bioactive composition of dandelion flowers and root and their health benefits, rather than of leaves (*2*).

The stability of bioactive compounds is a critical parameter for their successful incorporation in food products, since they are sensitive to environmental conditions (oxygen, light, heat and water), and therefore, their shelf life and bioavailability are affected. After oral consumption, bioactive compounds are going through rapid intestinal and first-pass metabolism, followed by transformation of their chemical structure and changes in bioactivities. Today, scientists are searching for some adequate solutions that will ensure stability of bioactive compounds in the gastrointestinal tract, allow their controlled release at the appropriate target in the organism and protect them during food processing and storage (*3*). Accordingly, encapsulation represents an effective technique that can ensure protection of a wide range of the specific sensitive compounds or whole extracts in adequate carrier systems. There are various techniques to encapsulate extracts: electrohydrodynamic process such as electrospinning and electrospraying (*4*-*6*), phase changes such as nanoprecipitation and antisolvent-dialysis (*7*, *8*) and spray drying (*9*, *10*).

Spray drying is a well-established encapsulation technique in a food sector, mainly employed to produce commercial micro-sized powders from liquid feedstocks in a single step (*11*). Around 90% of all industrially produced microencapsulated compounds are prepared by spray drying (*12*). Such wide application of spray drying could be ascribed to its numerous advantages: process simplicity and low operating costs, scale-up capability, control of particle size, shape and morphology, fast and energy-efficient technology, possibility of working with highly viscous feeds through preheating, applicability to both hydrophilic and hydrophobic food ingredients, design of particles with controlled release properties, high encapsulation efficiency and extended shelf life of the obtained powders, *etc*. (*10*).

The final powder properties after spray drying are influenced by the feed properties, feed flow rate, gas flow rate, aspiration ratio, inlet air temperature and outlet temperature (*13*). In general, spray drying implies high temperatures, where inlet air temperature is usually from 150 to 200 °C, while outlet temperature is usually around 70-90 °C (*9*). The application of high inlet temperature reduces the relative humidity of the drying gas and forms particles with less moisture content, leading to a drier powder that does not stick to the drying chamber (*14*). The outlet temperature is a result of the air inlet temperature, drying gas flow rate, feed flow rate and feed concentration (*9*). A higher inlet temperature causes a proportional increase in the outlet temperature (*15*). In order to obtain a powder with the low moisture content, it is preferable to achieve the small temperature difference between the inlet and outlet temperature and to set high inlet temperature (*9*). However, if the temperatures during spray drying are too high, degradation of sensitive and volatile bioactive compounds could occur, followed by reduced effectiveness of the encapsulation process (*16*). Although compounds are subjected to this temperature only for a short time, which should not affect much the bioactive properties of encapsulates (*3*), the application of low, both inlet and outlet, temperatures should be of interest for scientists and industry. Although many scientists are still using high inlet temperatures, some authors have applied low inlet temperatures (~100-130 °C) for spray drying of plant extracts, but they encountered certain challenges. These challenges were mainly related to a high moisture content of the powders after the application of low temperatures, which is not suitable for adequate shelf life of powders.

Along with the application of low inlet temperature that could highly protect sensitive bioactive compounds, the appropriate choice of suitable carrier in spray drying also plays a crucial role. Employed carriers could strongly determine the physicochemical and morphological properties of the produced microparticles, as well as the encapsulation efficiency of the entrapped compound. The most often used biopolymers for spray drying include natural gums, proteins, carbohydrates and lipids (*17*). Thus, to produce high quality spray drying powders with appropriate physicochemical, bioactive and sensory characteristics, many parameters should be considered.

Despite numerous health-promoting effects of dandelion and according to the authors’ knowledge, there are no many studies dealing with spray drying of dandelion extract. Only two studies focused on the encapsulation of dandelion bioactive compounds using ionic gelation technique. Bušić *et al.* (*18*) immobilized polyphenols from dandelion leaf extract using ionic gelation of alginate and implemented some new filler materials (cocoa powder and carob) to alginate network. On the other hand, Belščak-Cvitanović *et al.* (*19*) examined emulsion-templated microencapsulation of dandelion flower polyphenols using ionotropic gelation of alginate and pectin. Both approaches enabled high protection of dandelion polyphenols.

Thus, for the first time, this study aims to microencapsulate aqueous dandelion (*Taraxacum officinale* L.) leaf extract using spray drying and different carrier materials (guar gum, gum arabic, inulin, maltodextrin, pectin and alginate). With the aim of protecting sensitive dandelion polyphenols, low inlet temperature (130 °C) was employed. The influence of carrier type and their content on physicochemical, morphological and colour properties of the obtained dandelion powders was evaluated. The encapsulation efficiency of dandelion polyphenols (chicoric acid, total polyphenols and hydroxycinnamic acids), retained antioxidant capacity and release profiles of polyphenols and antioxidant capacity of dandelion powders in simulated gastrointestinal fluids were examined as well.

## MATERIALS AND METHODS

### Materials

Dandelion (dry leaves) was purchased at a local herbal store Suban Ltd. (Strmec Samoborski, Croatia). Guar gum and gum arabic were purchased from HiMedia Laboratories Private Ltd. (Mumbai, India). Maltodextrin (DE=16-19.9) and inulin (Cargill Inc., Wayzata, MN, USA) were generously donated by Palco Ltd. (Zagreb, Croatia). Pectin (Classic CU 902) was kindly donated by Herbstreith & Fox KG (Neuenbürg, Germany). Alginic acid sodium salt from brown algae (low viscosity) was obtained from Sigma-Aldrich, Merck (St. Louis, MO, USA). Folin-Ciocalteu reagent, sodium carbonate, *o*-phosphoric acid, hydrochloric acid, sodium chloride and sodium dihydrogen phosphate dihydrate were supplied by Kemika (Zagreb, Croatia). Sodium nitrite, sodium hydroxide and ethanol were purchased from Gram-mol d.o.o. (Zagreb, Croatia). Disodium hydrogen phosphate was provided by Fisher Chemical (Loughborough, UK). Gallic acid and HPLC standards, ABTS [2,2′-azino-bis(3-ethylbenzothiazoline-6-sulfonic acid)diammonium salt], Trolox (6-hydroxy-2,5,7,8-tetramethylchromane-2-carboxylic acid), potassium persulfate and sodium molybdate were obtained from Sigma-Aldrich, Merck (Steinheim, Germany). Methanol (HPLC grade) was supplied by J.T. Baker (Deventer, the Netherlands). Chemicals used for the HPLC analysis were HPLC grade, while others were of analytical grade.

### Preparation of dandelion extract and carrier solutions for spray drying

Dry dandelion leaves were ground using a domestic grinder Braun KSM2 (Kronberg, Germany) and sieved to obtain a homogenized fraction (~200 µm). Dandelion extracts were prepared by pouring 200 mL of distilled water (80 °C) over 20 g of plant and stirring with a glass rod for 30 min. The water temperature was maintained during the extraction. Afterwards, the obtained dandelion extract was filtered through a tea strainer containing a 4-layer cotton gauze and filled up with distilled water to 200 mL.

Carrier/delivery solutions for spray drying were prepared by dissolving different biopolymers in previously prepared dandelion extract (200 mL) containing (in %, *m*/*V*): guar gum 0.5, gum arabic, pectin and alginate 4, inulin and maltodextrin 10%. Selected viscosity of the carriers was chosen in order to provide adequate liquid atomization during spray drying and to collect free-flowing powders. The prepared carrier solutions were mixed overnight on a magnetic stirrer at 300 rpm (C-Mag HS 7; IKA, Staufen, Germany) and 4 °C, and then 200 g of each solution were spray dried.

### Microencapsulation by spray drying

Dandelion extract and carrier solutions were spray dried using a mini Büchi B-290 spray dryer (Büchi Labortechnik AG, Flawil, Switzerland), equipped with a 0.7 mm diameter nozzle. Laboratory-scale operating conditions were chosen according to preliminary tests and set as follows: inlet temperature (130±2) °C, outlet temperature (66±2) °C, air flow rate 600 L/h, liquid feed rate 8 mL/min (30%), aspiration 100%, compressed air for liquid atomization (600 kPa) and nozzle pressure drop 55 kPa. The obtained dandelion powders (one plain and six carrier-containing powders: guar gum, gum arabic, inulin, maltodextrin, pectin and alginate) were collected in tight plastic containers and stored at 4 °C until the analysis. Plain dandelion powder served as a control sample.

The process yield (%) of dandelion powder was calculated as the ratio of the total dry mass of the obtained powders (*m*_2_) and the dry mass of the material in its own carrier solution (*m*_1_), as follows (*20*):

Yield=(*m*_2_/*m*_1_)·100 /1/

### Physicochemical properties

The moisture content (%) of dandelion powder was determined gravimetrically by oven drying (oven Tehtnica, Železniki, Slovenia) at 105 °C to a constant mass, according to the modified standard AOAC Method 966.02 (*21*).

Solubility (%) of dandelion powder was determined according to the modified method described in a study of Belščak-Cvitanović *et al.* (*22*). A mass of 2.5 g of powder was suspended in 25 mL of distilled water at 30 °C. The suspension was stirred occasionally for 30 min and centrifuged (centrifuge SL 8R; Thermo Fisher Scientific, Suzhou, PR China) for 10 min at 10 867×*g*. The supernatant was collected, fully drained into an aluminium dish and dried to a constant mass at 105 °C. The mass of the solids recovered after drying was used to calculate the solubility of the powders.

Wettability of dandelion powder was measured using the modified method described by Jinapong *et al.* (*23*). Briefly, 0.1 g of powder was sprinkled into a 250-mL beaker and then 100 mL of distilled water (25 °C) were added. Wettability was expressed as a time in seconds that is required for all the powder to become completely wet (when all the particles penetrate the surface of the water).

For determination of bulk density (*ρ*_B_/(g/mL)), dandelion powder was gently loaded into a graduated cylinder and the exact mass of sample, along with the volume occupied by the sample, was recorded. The *ρ*_B_ was determined by dividing the net mass of the sample with the volume occupied by the sample in the cylinder. The tapped density (*ρ*_T_/(g/mL)) was calculated by dividing the net mass of the sample with the volume of sample in the cylinder after it was gently tapped 100 times onto an appropriate rubber mat.

Flowability and cohesiveness of dandelion powder were expressed through Carr index (CI, %) and Hausner ratio (HR) and calculated according to the following equations (*23*):

CI=(*ρ*_T_-*ρ*_B_/*ρ*_T_)·100 /2/

HR=*ρ*_T_/*ρ*_B_ /3/

Flowability of powders based on CI was classified as follows: <15 very good, 15-20 as good, 20-35 fair, 35-45 bad and >45 very bad. Cohesiveness based on HR was estimated in the following order: <1.2 low, 1.2-1.4 intermediate and >1.4 high (*23*).

The particle size distribution (PSD) of dandelion powders was measured by a laser diffraction method using Mastersizer 2000 (Malvern Instruments, Worcestershire, UK) equipped with the Scirocco 2000 dispersion unit. The PSD parameters included *d*(0.5) and span. Parameter *d*(0.5) is also known as the mass median diameter or the median of the volume distribution, and represents the size in µm at which 50% of the sample is smaller and 50% of the sample is larger than this size. The span represents the relative factor value which describes the PSD width. The span value was calculated according to the following equation:

Span=(*d*(0.9)-*d*(0.1))/*d*(0.5) /4/

where *d*(0.9) and *d*(0.1) are the diameters at which 90 and 10% of the population is below each value, respectively. As the span value is closer to 1, the PSD is narrower (*20*, *24*).

All analyses were performed in triplicate and presented as mean value±standard deviation (S.D.).

### SEM analysis

Scanning electron microscopy (SEM) analysis was applied to evaluate morphological characteristics of dandelion powders. The analysis was performed using a TESCAN Mira3 microscope (TESCAN ORSAY HOLDING a.s., Brno, Czech Republic). Powders were applied to stubs using a two-sided adhesive tape coated with a layer of gold (50 nm) and analysed at an acceleration voltage of 4-5 kV.

### Colour properties

The colour, i.e. L* (lightness), a* (redness and greenness) and b* (yellowness and blueness) values, of dandelion powders was evaluated using a spectrophotometer (CM-3500d; Konica Minolta, Tokyo, Japan). For the analysis, powders were put into adequate Petri dishes ensuring a homogenous and representative sample.

Total colour difference (*∆E*) was calculated according to the following equation (*25*):

*∆E*=√(L*_0_–L*)^2^+(a*_0_ –a*)^2^+(b*_0_ –b*)^2^ /5/

where subscript 0 refers to the colour values of plain dandelion powder (reference).

The colour deviation in comparison to the reference sample was rated according to the following range: Δ*E*<0.2 (no visible colour difference), Δ*E*=0.2-1.0 (noticeable colour difference), Δ*E*=1-3 (visible colour difference), Δ*E*=3-6 (well visible colour difference) and Δ*E*>6 (apparent colour deviation) (*26*). Five replicate measurements were performed and the results were presented as mean value±S.D.

### Determination of specific polyphenolic compounds

Specific polyphenols in dandelion powder were identified and qualified on Agilent 1100/1200 Series HPLC device, equipped with a photodiode array detector (Agilent, Santa Clara, CA, USA) and a reversed-phase column ACE Excel 5 SuperC18 (Advanced Chromatography Technologies, Aberdeen, Scotland, UK) (250 mm×4.6 mm, 5 μm i.d.). For the analysis, 0.25 g powder was dissolved in 10 mL distilled water until complete dissolution. In order to eliminate the polysaccharides, the solutions were mixed in a defined ratio with methanol, centrifuged (centrifuge SL 8/ 8R; Thermo Fisher Scientific) at 10 867×*g* for 10 min and filtered through 0.45-mm cellulose acetate filters (Nylon Membranes, Supelco, Bellefonte, PA, USA). After precipitation with methanol, 10 μL filtered sample were injected into the system. HPLC analysis was performed according to the method of Belščak-Cvitanović *et al.* (*27*), using *φ*(*o*-phosphoric acid)=0.1% in water or in methanol as solvents. Specific compounds were identified by comparing the retention times and spectral data with those of standards, while quantification was performed using calibration curve of each standard. Results were expressed as mg of identified compound per g of sample. Analyses were repeated three times and the results were presented as mean value±S.D.

### Encapsulation efficiency

The contents of chicoric acid (ChicA), total polyphenols (TP), hydroxycinnamic acids (HCA) and retained antioxidant capacity in dandelion powders were evaluated by dissolving 0.25 g powder in 10 mL distilled water with mixing on a magnetic stirrer (C-Mag HS 7; IKA) until complete dissolution. The encapsulation efficiency (%) was calculated as the ratio between the content of the investigated compound in the aqueous solution of dissolved powders and its respective content in the initial carrier solution. The content of ChicA was determined by HPLC as previously explained in the section *Determination of specific polyphenolic compounds.* TP were determined spectrophotometrically (model Helios γ; ThermoSpectronic, Cambridge, UK) according to the modified method of Lachman *et al.* (*28*), using Folin-Ciocalteu's reagent, while HCAs were evaluated in a reaction with Arnow reagent (*29*). Antioxidant capacity was measured with the ABTS radical cation decolorization assay (*30*). Analyses were repeated three times and the results were presented as mean value±S.D.

### Fourier-transform infrared spectroscopy

The attenuated total reflectance Fourier-transform infrared spectroscopy (ATR-FTIR) analysis of plain carriers, obtained plain dandelion powder and carrier-containing dandelion powders was performed using the IRAffinity-1 FTIR spectrophotometer (Shimadzu, Kyoto, Japan). The spectral range was from 4000 to 600 cm^-1^, while the resolution was 4 cm^-1^.

### In vitro release of polyphenols and antioxidant activity

The release profiles of TP, HCA and antioxidant capacity (ABTS assay) of the obtained dandelion powders were determined in a simulated gastric (SGF) and intestinal (SIF) fluids. SGF consisted of sodium chloride and hydrochloric acid (pH=1.2), while SIF comprised phosphate buffer (pH=7.4). For the analysis, 0.3 g powder was suspended in 30 mL SGF, previously heated at 37 °C, and mixed for 120 min on a magnetic stirrer (C-Mag HS 7; IKA) at 100 rpm. The suspension was kept constantly at 37 °C. At defined time intervals, an aliquot of 2 mL was withdrawn from the solution and shortly centrifuged in a mini centrifuge (MLX-106; Life Science Products, Inc., Frederick, CO, USA) at 2000×*g*. The clear supernatant was collected for the analysis. An aliquot of the fresh fluid (2 mL, 37 °C) was added back to the solution just after the supernatant was taken out. After 120 min in SGF, the solution was centrifuged (centrifuge SL 8/8R; Thermo Fisher Scientific) at for 5 min 10 867×*g* and the supernatant consisting of SGF was discarded. Collected microparticles were washed with distilled water and suspended in 30 mL SIF, previously heated at 37 °C, at previously defined conditions (37 °C, 100 rpm). Samples were again collected at defined time intervals, as described, until crude microparticles were completely disintegrated (in 240 min). The release profile was determined by evaluating the content of TP (mg gallic acid equivalents, GAE per g of sample), HCA (mg caffeic acid, CaffA per g of sample) and antioxidant capacity (mmol Trolox per g of sample), as described in section *Encapsulation efficiency of polyphenols and retained antioxidant capacity*, in SGF/SIF supernatants collected at a defined time. Analyses were repeated twice and the results were presented as mean value±S.D.

### Statistical analysis

Statistical analysis was performed using Statistica, v. 12 (*31*), where one-way analysis of variance (ANOVA) with the Tukey’s *post hoc* test was run to determine the influence of different carrier materials and their content on the physicochemical and colour properties, polyphenolic content and encapsulation attributes of dandelion powders. The probability level of p<0.05 was considered significant.

## RESULTS AND DISCUSSION

The results of the process yield obtained after spray drying of carrier solutions are not shown in a table. However, the yield was obtained in the following descending order (in %): gum arabic-dandelion powder 86.82>maltodextrin-dandelion powder 75.15>inulin-dandelion powder 74.74>guar gum-dandelion powder 68.80>pectin-dandelion powder 54.75>alginate-dandelion powder 48.50.

### Physicochemical properties of dandelion powders produced by spray drying

The moisture content of plain dandelion powder was 6.1% (Table 1), and this sample was insignificant (p>0.05) only to gum arabic-dandelion powder. The addition of carriers to the delivery solution differently affected moisture content. Implementation of gum arabic, inulin and maltodextrin in the delivery solution decreased moisture content of the evaluated dandelion powder, while guar gum, pectin and alginate increased its values. The lowest moisture content (1.93%) was achieved in maltodextrin-dandelion powder, which differed significantly (p<0.05) from all other samples. Guar gum-dandelion powder exhibited the highest moisture content (8.0%), insignificantly (p>0.05) different from the values obtained for pectin-dandelion powder (7.60%) and alginate-dandelion powder (7.3%). These results were expected, since maltodextrin-dandelion powder had the highest content of the carrier (10%) in the delivery solution and consequently the highest total solid content. Contrary was noted for guar gum-dandelion powder (0.5% of carrier). The results indicated a higher influence of carrier content on the moisture content of dandelion powder, rather than carrier type.

**Table 1:** Physicochemical properties of plain dandelion powder and carrier-containing dandelion powders produced by spray drying

Dandelion powder	*w*(moisture)/%	Solubility/%	Wettability/s	*ρ*_B_/(g/mL)	Carr index/%	Hausner ratio	*d*(0.5)/µm	Span (PSD)
Plain	(6.1±0.5)^a^	(91.8±0.5)^a^	(238.0±0.8)^ac^	(0.28±0.01)^a^	(49.7±2.7)^a^	(2.0±0.1)^a^	(71.1±2.2)^a^	(4.3±0.2)^a^
Guar gum	(8.0±0.6)^bf^	(97.1±0.8)^b^	(27 601±98)^b^	(0.20±0.01)^b^	(44.0±1.3)^bghi^	(1.81±0.07)^bhij^	(22.4±1.7)^bh^	(12.9±0.6)^b^
Gum arabic	(5.25±0.00)^ac^	(92.5±0.6)^aef^	(434.0±9.0)^a^	(0.29±0.01)^a^	(41.08±0.03)^cgjk^	(1.70±0.00)^chkl^	(10.31±0.01)^ci^	(28.10±0.03)^c^
Inulin	(4.8±0.1)^c^	(92.2±0.3)^ag^	(218.0±5.7)^ae^	(0.25±0.01)^cgh^	(37.9±0.2)^djl^	(1.61±0.00)^dkmn^	(7.1±0.2)^djk^	(3.24±0.06)^dh^
Maltodextrin	(1.93±0.02)^d^	(92.0±0.6)^a^	(281.0±8.2)^af^	(0.25±0.00)^dgi^	(46.2±1.5)^ah^	(1.86±0.05)^ei^	(6.7±0.2)^ejl^	(2.79±0.07)^ehi^
Pectin	(7.60±0.06)^ebg^	(93.98±0.09)^cbe^	(98.0±3.3)^cef^	(0.24±0.01)^ehi^	(41.8±1.2)^eik^	(1.72±0.04)^fjlm^	(22.3±1.0)^fh^	(8.7±0.2)^f^
Alginate	(7.3±0.4)^fg^	(95.4±0.4)^dbfg^	(24 218±165)^d^	(0.39±0.00)^f^	(35.7±0.0)^fl^	(1.56±0.00)^gn^	(7.98±0.02)^gikl^	(2.16±0.01)^gi^

PSD=particle size distribution. The values in the same column with a different letter in superscript indicate significant (p<0.05) differences among samples. Values are expressed as mean±S.D. (*N*=3)

In the present study, to preserve the stability of sensitive polyphenols during spray drying, low inlet temperature (130 °C) was applied. In general, the usage of high inlet temperatures (150-190 °C, and higher) leads to the faster heat transfer between the product and the drying air. Consequently, a higher temperature gradient is achieved between the atomized feed and the drying air, resulting in the greatest driving force for the water evaporation and reduced moisture content of powders (*32*). This was confirmed by Sablania and Den Bosco (*33*), who reported decreased moisture content of powders after increasing inlet temperature. Moreover, authors found inlet temperature of 165 °C as optimal for spray drying of *Murraya koenigii* leaf extract using maltodextrin and gum arabic, with moisture content of 3.03%. Bhusari *et al.* (*34*) used 180 °C for spray drying of tamarind pulp powder in maltodextrin, gum arabic and whey protein concentrate. They determined the moisture content in a range of 3.65−7.11%. The results of both studies are similar to the ones obtained for the moisture content in this study, despite the fact that these authors used much higher inlet temperatures. Thus, the present results justify the application of inlet temperature of 130 °C, indicating that implementation of low temperatures could also result in powders with appropriate and desirable moisture content, but also considering preservation of sensitive bioactive compounds.

There are many factors that could influence the solubility of spray-dried powders: properties of raw materials, carrier systems (type and content), physicochemical properties of the final powder (moisture content, particle size, physical form of the particle) and drying parameters (atomization, inlet and outlet temperatures and feed flow rate) (*35*). The solubility of the obtained dandelion powders was higher than 90%, which is in agreement with other studies (*20*, *36*). It ranged from 91.8 (plain dandelion powder) to 97.1% (guar gum-dandelion powder) (Table 1). The obtained results implied that the presence of carriers enhanced the solubility of dandelion powder, especially in the case of guar gum-, pectin- and alginate-dandelion powders. These samples had significantly (p<0.05) higher solubility than plain dandelion powder. Among carrier-containing dandelion powders, the one containing maltodextrin was characterized by the lowest solubility (92.0%), which was significantly (p<0.05) lower than the solubility of dandelion powders containing guar gum, pectin or alginate. Moreover, samples containing the highest carrier content (10%, maltodextrin- and inulin-dandelion powders) had the lowest solubility. On the other hand, the sample prepared with the lowest carrier content (0.5%, guar gum-dandelion powder) was characterized by the highest solubility. This highlighted the impact of carrier content on the solubility of dandelion powders. However, when observing carrier-containing dandelion powders prepared with the same carrier content (groups prepared with 4 or 10%), insignificant (p>0.05) difference within each group was reported.

The selected delivery materials differently affected the wettability of the produced dandelion powders. Compared to plain dandelion powder, there was an uneven trend after the addition of carriers to the delivery mixture. Plain dandelion powder became completely wet after 238 s and it was significantly (p<0.05) different from guar gum- and alginate-dandelion powders. The longest time to penetrate the surface of the water was determined for dandelion powders containing guar gum (27 601 s) and alginate (24 218 s) (Table 1). These samples differed significantly (p<0.05) from each other and from all other carrier-containing dandelion powders. The shortest time to get wet was measured for pectin-dandelion powder (98 s), whose values of wettability were significantly (p<0.05) different from guar gum-, gum arabic- and alginate-dandelion powders. An uneven trend was observed in the influence of carrier content on the wettability of the prepared powders. Thus, the obtained results indicated that the wettability of powders was more influenced by the carrier type, rather than the carrier content.

The bulk density of plain dandelion powder (0.28 g/mL) differed significantly (p<0.05) from other powders, except for gum arabic-dandelion powder (0.29 g/mL) (Table 1). Alginate-dandelion powder had significantly (p<0.05) higher bulk density (0.39 g/mL) than the other samples. Bhusari *et al.* (*34*) determined similar bulk density values (0.39−0.69 g/mL), where the bulk density of tamarind pulp powders decreased after increasing the carrier content. In the present study, the sample prepared with the lowest carrier content (0.5%, guar gum-dandelion powder) was characterized by the significantly (p<0.05) lowest bulk density value (0.20 g/mL), since it differed significantly (p<0.05) from all other samples. These results could be related to the moisture content of guar gum-dandelion powder, since this sample contained the highest moisture content. However, the same trend was not observed for samples containing the highest carrier content (10%, maltodextrin- and inulin-dandelion powders). These samples were not marked with the highest values of bulk density. An uneven trend, depending on the carrier content, was observed in the study of Şahin-Nadeem *et al.* (*37*). Moreover, Belščak-Cvitanović *et al.* (*16*) determined that the content of carriers did not show marked influence on the bulk density of green tea powders. On the other hand, the type of carrier exhibited significant effect on the bulk density of powders. The present study also revealed the main impact of carrier type rather than its content on the bulk density of dandelion powders.

Carr index (CI), a flowability parameter, ranged from 35.7 (alginate-dandelion powder) to 49.7% (plain dandelion powder) (Table 1). Compared to plain dandelion powder, CI of carrier-containing powders decreased, indicating enhanced flowability properties of the dandelion powder after the addition of carriers to the delivery solution. The flowability of plain and maltodextrin-dandelion powders was characterized as very bad (CI>45), and these samples were insignificantly (p>0.05) different from each other. The flowability of other samples was ranked as bad (CI=35−45).

Hausner ratio (HR), a parameter indicating cohesiveness of the produced powders, followed the results obtained for CI. HR of carrier-containing dandelion powders (1.56−1.86) was lower than the HR of plain dandelion powder (2.0), which differed significantly (p<0.05) from all carrier-containing dandelion powders (Table 1). If HR is lower, the cohesiveness of powders is more favourable. Even though HR values of carrier-containing dandelion powders decreased, which is favourable, all samples showed high cohesiveness (HR>1.4).

When observing the delivery system with the best flow and cohesive properties, the one prepared with alginate could be highlighted as the best, due to the lowest CI and HR values. The content of carriers differently affected CI and HR, since the established trend cannot be seen. Here, this put the importance of choosing a suitable carrier in the first place.

PSD parameters for the examined dandelion powders are presented in terms of *d*(0.5) and PSD width (span) and they are shown in Table 1. Particle size is one of the most important quality parameters that is determined in the industry of food powders, since it could highly affect their handling, transportation and shelf life properties (*35*). It is related to the atomizer type, physical properties of the carrier solution and content (*38*). In the present study, *d*(0.5) of plain dandelion powder was 71.1 µm. On the other hand, *d*(0.5) of carrier-containing dandelion powders was significantly (p<0.05) lower, even 3−10 times, and it ranged from 6.7 to 22.4 µm (Table 1). This highlighted the importance of carriers in the delivery solutions. In general, a small particle size of around <50 µm is characteristic of spray-dried powders (*39*), which is consistent with the results obtained here. Among carrier-containing dandelion powders, the smallest *d*(0.5) was characteristic of maltodextrin-dandelion powder, while the largest *d*(0.5) was of guar gum-dandelion powder. These results suggested that samples prepared with a higher content of carrier (10%, maltodextrin and inulin, insignificant (p>0.05) difference) resulted in powders with smaller particle size, and *vice versa*. Such results again underlined the impact of carrier content, rather than the carrier type on the *d*(0.5) of dandelion powders. Hashib *et al.* (*40*) also reported this relation, where the particle size of spray-dried pineapple powders decreased after increasing the content of maltodextrin. However, there are differences among studies including the PS and the carrier content. Contrary to previous results, some studies reported that increasing the carrier content in the delivery solution leads to a larger particle size (*41*).

Furthermore, the powder particle size could influence some other physical properties, like the bulk density. In general, the bulk density of powders increases with decreasing particle size, since particles with smaller size reduce the void spaces among particles and put the particles in close form (*42*). This is consistent with the present results. Such relation was observed for alginate-dandelion powder, which had the highest bulk density (0.39 g/mL) and the smallest *d*(0.5) (7.98 µm). Conversely, guar gum-dandelion powder had the lowest bulk density (0.20 g/mL) and the highest *d*(0.5) (22.4 µm).

Span factor values were calculated employing *d*(0.1), *d*(0.5) and *d*(0.9) values, according to Eq. 4. The span of plain dandelion powder (4.3) was significantly (p<0.05) different from all other samples, and its value, depending on the employed carrier, was lower or higher (Table 1). Among carrier-containing dandelion powders, samples prepared with gums exhibited the highest span values (guar gum-dandelion powder 12.9 and gum arabic-dandelion powder 28.10), but their values were significantly different (p<0.05). On the contrary, the lowest and insignificant (p>0.05) span values were determined for alginate- (2.16) and maltodextrin-dandelion powders (2.79). Since span values of all dandelion powders were higher than 1, the obtained results implied that samples analysed in this study indicate broader size distribution and high polydispersity of spray-dried powders. However, this is not surprising for spray-dried particles. Ćujić-Nikolić *et al.* (*43*) also reported span values higher than 1 for spray-dried chokeberry extracts, similar to this study.

When observing the influence of carrier content on PSD width, an uneven trend was reported, indicating here the greater importance of the carrier type, rather than its content.

### SEM analysis of dandelion powders produced by spray drying

**Fig. 1:**
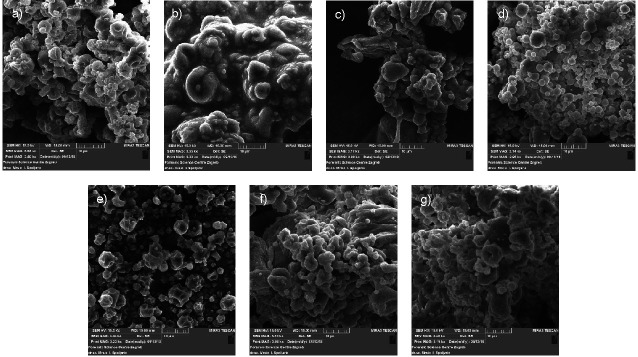
Scanning electron microscopy micrographs of: a) plain dandelion powder and dandelion powders containing: b) guar gum, c) gum arabic, d) inulin, e) maltodextrin, f) pectin and g) alginate produced by spray drying

SEM micrograph of plain dandelion powder revealed polydispersed distribution of the microparticles, with dented surface and visible cracks (Fig. 1a). The improvement of morphological properties of carrier-containing dandelion powders highly depended on the carrier selection (Fig. 1).

When using hydrocolloid gums, like guar gum and gum arabic (Figs. 1b and 1c), an extremely inhomogeneous structure was still observable. The particles were not finely dispersed, revealing irregular and highly compacted structure of these powders. Certain bulges on the surface of guar gum-dandelion powder were also visible. The most favourable morphology among the samples was determined for inulin-dandelion powder (Fig. 1d). This sample was characterized by the most spherically shaped and uniform microparticles, smooth surface and no visible dents and ruptures on the surface. When maltodextrin was used as a carrier (Fig. 1e), wrinkled shape, shrinking and structure cracking were observed. However, the sphericity of the microparticles of maltodextrin-dandelion powder was somewhat improved compared to the powders prepared with guar gum and gum arabic. Araujo-Díaz *et al.* (*44*) reported similar morphology when employing inulin and maltodextrin as carriers for spray drying of blueberry juice. They also obtained particles with a spherical shape and smooth surface when using inulin, while rougher surface was determined in maltodextrin powders, which is in accordance with this study. Furthermore, compared to maltodextrin-dandelion powder, similar morphological characteristics were obtained of pectin- and alginate-dandelion powders (Figs. 1f and 1g). However, less rough surface and collapsed form, but with a more irregular particle shape were observed for these two samples.

In addition, when observing SEM images of carrier-containing dandelion powders, inulin- and maltodextrin-dandelion powders revealed the most homogeneous and loose structure of the microparticles, with minimal agglomeration. This could be in correlation with particle size, since these samples scored the lowest *d*(0.5). However, if correlating the morphology of the powders with the carrier content, an uneven trend is observable. Thus, the obtained results showed a greater importance of the selected carrier type than its content on the morphology of dandelion extract.

### Colour properties of dandelion powders produced by spray drying

Whether spray-dried powders are used as final products or they are intended for implementation in some other products, the determination of the colour properties is one of the most important quality parameters, especially if taking into account that coloured powders can be used as colouring agents.

The lightness (L*) of plain dandelion powder was 57.2 (Table 2). The addition of carriers to the delivery solution significantly (p<0.05) increased L* values, resulting in a lighter colour of these samples. The exception was guar gum-dandelion powder, which was insignificantly (p>0.05) darker (L*=56.7) than the plain one (lower L* value). Among carrier-containing dandelion powders, the powder containing maltodextrin was screened as the lightest one (L*=81.3), significantly (p<0.05) different from others. When observing the impact of carrier content on L* values, the lightness of samples increased after increasing the carrier content. Thus, dandelion powders containing 10% maltodextrin or inulin were the lightest. On the contrary, the sample prepared with the lowest content of the carrier (0.5% guar gum) was the darkest, suggesting strong effect of the carrier content on the lightness of the evaluated powders. Also, an important impact of the carrier type on the lightness of dandelion powders was observed, since all samples prepared with an equal content of carrier were significantly (p<0.05) different. These results suggest that both the carrier type and its content have a strong effect on the lightness of dandelion powders.

**Table 2:** Colour properties of plain dandelion powder and carrier-containing dandelion powders produced by spray drying

Dandelion powder	L*	a*	b*	*∆E*
Plain	(57.2±0.3)^a^	(5.6±0.1)^a^	(28.6±0.2)^a^	-
Guar gum	(56.7±0.2)^a^	(7.81±0.04)^b^	(30.08±0.01)^b^	(2.70±0.07)^a^
Gum arabic	(70.8±0.2)^b^	(2.58±0.05)^ch^	(21.0±0.3)^ch^	(15.89±0.01)^b^
Inulin	(79.4±0.8)^c^	(1.3±0.1)^d^	(19.1±0.3)^d^	(24.6±0.9)^c^
Maltodextrin	(81.3±0.3)^d^	(0.68±0.05)^e^	(16.8±0.4)^e^	(27.3±0.1)^d^
Pectin	(63.9±0.3)^e^	(4.20±0.03)^f^	(27.2±0.1)^f^	(7.0±0.2)^e^
Alginate	(69.2±0.5)^f^	(2.41±0.05)^gh^	(21.3±0.2)^gh^	(14.5±0.4)^f^

*∆E*=total colour difference. The values in the same column with a different letter in superscript indicate significant (p<0.05) differences among samples. Values are expressed as mean±S.D. (*N*=5)

Carrier-containing dandelion powders were significantly (p<0.05) greener (lower a* values) than plain dandelion powder. The exception was dandelion powder containing guar gum, with significantly (p<0.05) higher a* value than others, indicating a redder colour of this sample (Table 2). Among carrier-containing dandelion powders, the greenest was the one with maltodextrin (significantly (p<0.05) different from others), in descending order followed by (less green): inulin>alginate>gum arabic>pectin>guar gum. Such order revealed that the green colour of powder was stronger as the content of the carrier was higher and *vice versa*.

Compared to plain dandelion powder, the yellowness (b* values) significantly (p<0.05) increased in the guar gum-dandelion powder, while in other carrier-containing dandelion powders this value significantly (p<0.05) decreased. In general, as the powders were greener, their colour was less yellow. As mentioned above, maltodextrin- and inulin-dandelion powders were the greenest and accordingly they were the least yellow (the lowest b* values). However, maltodextrin- and inulin-dandelion powders had significantly (p<0.05) different values. The opposite was observed for the guar gum-dandelion powder, which was the least green, and accordingly its yellowness was the highest (Table 2). Thus, here the yellow colour of powder was stronger as the carrier content was lower.

Also, when observing both a* and b* values, an inverse relation with the applied carrier content was reported (higher content of carrier, lower a* and b* values). Şahin-Nadeem *et al.* (*37*) also reported the same decrease in a* and b* values of spray-dried sage powders after increasing the carrier content, while L* values increased, as it was the case in this study. Moreover, gum arabic- and alginate-dandelion powders (4% carrier) exhibited insignificant difference (p>0.05) in terms of both a* and b* values, while other samples prepared with the same carrier content were significantly (p<0.05) different. In general, if taking into account the results obtained for a* and b* values of dandelion powders, both the carrier type and content had a high impact on the greenness and yellowness of the examined powders.

Since L*, a* and b* values could be changeable each time due to the nature of raw material, it is important to observe colour differences in terms of *∆E*. The lowest *∆E* (2.70) was ascribed to guar gum-dandelion powder, while maltodextrin-dandelion powder scored the highest *∆E* (27.3), as shown in Table 2. The results indicated that as the content of carrier is higher, *∆E* is higher as well, and reciprocally. Furthermore, *∆E* among all carrier-containing dandelion powders was significantly (p<0.05) different. These results followed the ones determined for other colour parameters, where both the carrier type and content had high influence on *∆E* of dandelion powders. According to the determined *∆E* values, visible colour difference was reported for guar gum-dandelion powder, while other powders were characterized by an apparent colour difference.

### Polyphenolic composition, encapsulation efficiency of polyphenols and retained antioxidant capacity of dandelion powders produced by spray drying

HPLC analysis of dandelion powders revealed HCAs as a major group of polyphenolics found in dandelion leaves. Among them, caftaric (CaftA), chlorogenic (ChlA), caffeic (CaffA) and chicoric acid (ChicA) were identified in the analysed samples. Such superiority of HCAs among other polyphenolic groups was conﬁrmed in other studies, with chicoric acid representing the main compound found in dandelion (*1*, *18*). In the present study, ChicA was also marked as the most abundant specific polyphenol of dandelion powder, followed by CaftA>ChlA>CaffA.

As shown in Table 3, the mass fraction of CaftA ranged from 2.03 (inulin-dandelion powder) to 10.50 mg/g (plain dandelion powder). When comparing plain dandelion powder and other carrier-containing powders, the latter had significantly (p<0.05) lower mass fraction of CaftA. This trend was also seen among other detected HCAs. This behaviour indicated that the addition of carriers to the delivery solution decreased the HCA content. Belščak-Cvitanović *et al.* (*16*) observed similarly that the contents of epigallocatechin gallate and caffeine decreased in green tea powders containing various biopolymers, compared to plain green tea powder. The results obtained in this study could be explained by the potential formation of polyphenol-polysaccharide complexes. Here, a certain amount of dandelion polyphenols could be attached to and absorbed on the used polysaccharide carrier, which eventually resulted in a reduced content of the detected free dandelion polyphenols. Namely, polyphenols are highly reactive molecules that can establish interactions with different macromolecules and consequently form different polyphenol-macromolecule complexes (*45*). Studies modelling plant cell walls using cellulose and pectin showed that such materials were able to absorb model phenolic acids and anthocyanins through non-covalent interactions (*46*). Moreover, guar gum-dandelion powder had the highest mass fraction of CaftA (9.21 mg/g) among carrier-containing dandelion powder, differing significantly (p<0.05) from the others. This pattern was also observed for other analysed HCAs. On the other hand, inulin-dandelion powder had the lowest mass fraction of CaftA (2.03 mg/g), also differing significantly (p<0.05) from the others, with the exception of maltodextrin-dandelion powder.

**Table 3:** The mass fraction of specific polyphenolic compounds, encapsulation efficiency of polyphenols and retained antioxidant capacity of plain dandelion powder and carrier-containing dandelion powders produced by spray drying

Dandelion powder	*w*/(mg/g)				EE/%			RAC/%
CaftA	ChlA	CaffA	ChicA	ChicA	TP	HCA
Plain	(10.50±0.02)^a^	(3.38±0.00)^a^	(1.12±0.00)^a^	(39.3±0.1)^a^	-	-	-	-
Guar gum	(9.21±0.01)^b^	(2.32±0.01)^b^	(0.73±0.01)^b^	(33.91±0.02)^b^	(55.9±0.1)^a^	(35.38±0.02)^a^	(42.80±0.00)^a^	(37.8±0.5)^a^
Gum arabic	(3.48±0.00)^c^	(0.39±0.02)^chi^	(0.15±0.00)^ch^	(12.01±0.02)^c^	(49.1±1.4)^b^	(42.12±0.04)^bg^	(58.99±0.01)^b^	(53.2±0.1)^b^
Inulin	(2.03±0.02)^dh^	(0.40±0.01)^dhj^	(0.11±0.00)^di^	(7.74±0.00)^dh^	(23.21±0.04)^cg^	(17.23±0.00)^ch^	(18.62±0.00)^c^	(21.5±0.1)^c^
Maltodextrin	(2.05±0.00)^eh^	(0.43±0.00)^eij^	(0.12±0.00)^ei^	(7.77±0.01)^eh^	(24.11±0.06)^dg^	(16.87±0.02)^dh^	(20.03±0.00)^d^	(24.5±0.5)^d^
Pectin	(5.9±0.2)^f^	(1.64±0.05)^f^	(0.33±0.02)^f^	(22.4±1.1)^f^	(74.4±3.8)^e^	(63.57±0.00)^e^	(67.90±0.00)^e^	(62.9±0.4)^e^
Alginate	(2.97±0.00)^g^	(0.64±0.01)^g^	(0.15±0.00)^gh^	(9.01±0.01)^g^	(39.17±0.02)^f^	(43.20±0.00)^fg^	(42.86±0.02)^a^	(40.1±0.2)^f^

CaftA=caftaric acid, ChlA=chlorogenic acid, CaffA=caffeic acid, ChicA=chicoric acid, TP=total polyphenols, HCA=hydroxycinnamic acids, EE=encapsulation efficiency, RAC=retained antioxidant capacity determined by ABTS assay. The values in the same column with a different letter in superscript indicate significant (p<0.05) differences among samples. Values are expressed as mean±S.D. (*N*=3)

The mass fraction of ChlA in plain dandelion powder was 3.38 mg/g, while among carrier-containing dandelion powders its mass fraction ranged from 0.39 (gum arabic-dandelion powder) to 2.32 mg/g (guar gum-dandelion powder) (Table 3). The mass fraction of ChlA in gum arabic-, inulin- and maltodextrin-dandelion powders was insignificantly (p>0.05) different from each other.

Furthermore, CaffA was reported as the least abundant (0.11−1.12 mg/g) HCA found in dandelion powders, while ChicA was as the most represented one (7.74−39.3 mg/g) (Table 3). As in other cases, plain dandelion powder had the highest mass fraction of both CaffA and ChicA, differing significantly (p<0.05) from the others. Among carrier-containing dandelion powders, the one with inulin had the lowest mass fraction of both acids, and this sample was insignificantly (p>0.05) different only from maltodextrin-dandelion powder. Guar gum-dandelion powder had the highest mass fraction of CaffA and ChicA, and it was significantly (p<0.05) different from all others.

In general, the results suggested an inverse relation of the carrier content and HCA mass fraction in dandelion powders, since the sample prepared with the lowest carrier content (0.5% guar gum) had the highest mass fraction of the analysed HCA, and reciprocally. This highlighted the impact of carrier content on HCA mass fraction. Siacor *et al.* (*47*) obtained similar trend, where polyphenol content in spray-dried mango powders highly decreased by increasing the carrier content. Such relation in this study could be ascribed to the possible interactions of dandelion polyphenols and employed carriers, where a higher carrier content could mean more material to interact with polyphenols, more polysaccharide-polyphenol complexes and therefore fewer free polyphenols to examine. Furthermore, samples prepared with the highest carrier content (10% maltodextrin or inulin) did not differ significantly (p>0.05) in the evaluated HCA mass fraction. On the other hand, gum arabic-, pectin- and alginate-dandelion powders prepared with 4% of carrier differed significantly (p<0.05) in the HCA mass fraction (exception being CaffA content in gum arabic- and alginate-dandelion powders). This revealed that the carrier type also affected the HCA mass fraction. The obtained results suggested that both the carrier type and content influenced the mass fraction of HCA in dandelion powders.

Since ChicA was the most abundant polyphenol in dandelion leaves, its encapsulation efficiency was examined using HPLC analysis. The highest encapsulation efficiency, and significant (p<0.05) to others, was evaluated in pectin-dandelion powder, with 74.4% ChicA entrapment (Table 3). It was followed by guar gum-dandelion powder (55.9%), while samples containing inulin (23.21%) and maltodextrin (24.11%) enabled the lowest ChicA retention. These two samples were insignificantly (p>0.05) different from each other. Considering that guar gum- and pectin-dandelion powders had the highest mass fraction of ChicA, while maltodextrin- and inulin-dandelion powders had the lowest one, such results are not surprising.

The results (spectrophotometrically determined) obtained for the encapsulation efficiency of TP confirmed pectin-dandelion powder as a sample with the highest entrapment rate of TP (63.57%), differing significantly (p<0.05) from the others. The encapsulation efficiency of around 40% was quantified for alginate- and gum arabic-dandelion powders, and it was not significantly different (p>0.05). The same insignificant trend (p>0.05) was determined for inulin- and maltodextrin-dandelion powders, which had the lowest encapsulation efficiency (~17%).

Similar results were obtained for the encapsulation efficiency of HCA, where again pectin-dandelion powder had the highest ability to encapsulate HCA (67.90%), significantly (p<0.05) higher than others (Table 3). Insignificant (p>0.05) difference in the encapsulation efficiency of HCA was obtained only for guar gum- and alginate-dandelion powders. On the other hand, inulin- (18.62%) and maltodextrin-dandelion powders (20.03%) showed the lowest ability to entrap HCA, but in this case they differed significantly (p<0.05).

ABTS analysis again highlighted pectin-dandelion powder as a sample with the highest ability (62.9%), while inulin- and maltodextrin-dandelion powders had the lowest ability (21.5−24.5%) to retain antioxidant capacity (Table 3). Also, all samples were significantly (p<0.05) different. Since mainly polyphenols are responsible for antioxidant capacity, such results that followed the ones determined for encapsulation efficiency of polyphenols are expected.

Moreover, it can be observed that samples prepared with the highest carrier content (10% maltodextrin or inulin) were the ones with the lowest ability to encapsulate polyphenolics and retain antioxidant capacity. On the contrary, that was not the case with the sample prepared with the lowest carrier content (0.5% guar gum), since this sample was not marked as the one with the highest encapsulation efficiency. Here, the group that contained 4% carrier (gum arabic, pectin and alginate) stood out as the group that enabled the highest encapsulation efficiency of polyphenolics and retained antioxidant capacity, with a pectin-dandelion powder being the most efficient. Thus, the results revealed a higher influence of the selected carrier type than its content on the encapsulation parameters of dandelion powders. In addition, there are certain discrepancies among the studies related to the encapsulation efficiency and carrier content. Arepally and Goswami (*48*) reported that the encapsulation efficiency of probiotics in spray drying increased after increasing the content of gum arabic. On the contrary, Şahin-Nadeem *et al.* (*37*) found that the encapsulation efficiency of sage TP notably decreased after increasing the carrier content, like in this study.

### FTIR spectroscopy of dandelion powders produced by spray drying

**Fig. 2:**
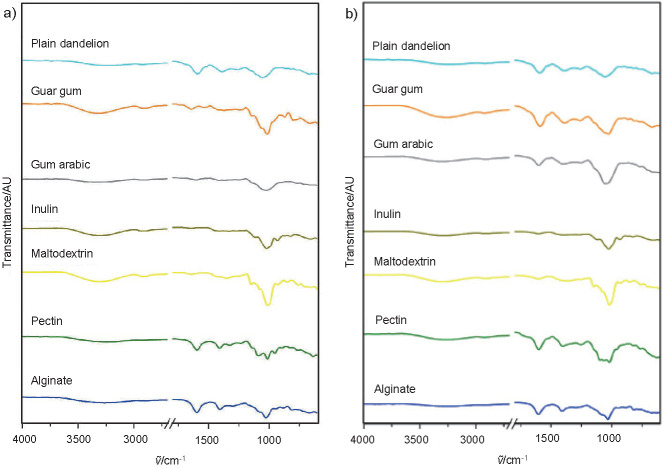
Fourier-transform infrared spectra of: a) plain carriers, and b) plain dandelion powder and carrier-containing dandelion powders produced by spray drying

The chemical properties of plain carrier materials, plain dandelion powder and carrier-containing dandelion powders were investigated using ATR-FTIR spectroscopy (Fig. 2). Sample spectra dominantly exhibited the bands that are ascribed to characteristic vibrations of carbohydrates ~1020 cm^-1^ (C-O stretching vibrations) and ~1410 cm^-1^ (-CH_2_ bending vibrations), while the band at around 1600 cm^-1^ is identified in the spectra of natural gums (*49*). Also, the spectra showed the presence of the O-H groups (~3000-3600 cm^-1^), and C-H vibrations were identified at ~2900 cm^-1^ (*16*, *19*).

It should be pointed out that during the preparation of initial delivery solutions, no visible interactions (*e.g.* precipitation) between ingredients were observed. This enabled satisfactory liquid flow and atomization during spray drying. Potential interactions between dandelion bioactive compounds and carriers may be observed by shifting in the position of the bands related to the O-H vibrations. After spray drying, the bands in the spectrum of guar gum-dandelion powders overlapped with the dandelion bands, indicating higher amounts of dandelion extract attached to the surface of particles and consequently suggesting a lower protection of the extract when this carrier was applied. Primary reason for this might be that the content of guar gum (0.5%) was the lowest among the employed carriers, which led to insufficient formation of a protective carrier layer around the extract. However, in some cases the usage of a higher carrier content is impossible, due to the high viscosity of their solutions that cannot be processed by spray drying. In the case of pectin-dandelion powder, overlapping of carrier bands around 1200−800 cm^-1^ was identified, suggesting that not only the content, but also the carrier type, is critical for optimal protection of the encapsulated compound. On the other hand, the domination of bands of gum arabic, inulin, maltodextrin and alginate in the spectra of corresponding carrier-containing dandelion powders possibly indicated relatively good protection of dandelion polyphenols by these carriers (*50*). Furthermore, in order to obtain high encapsulation efficiency and protection of active compounds, we selected carrier materials that are well established as suitable for this type of encapsulates and active ingredients. Chemical interactions between the carrier and the active compounds should be limited in order to facilitate all steps during encapsulation. Potential chemical interactions between the extract components and carriers are most probably molecular interactions, which may explain the changes in the positions of OH bands in the spectra of carrier-containing dandelion powders.

### Release profiles of polyphenols and antioxidant capacity of dandelion powders produced by spray drying

**Fig. 3:**
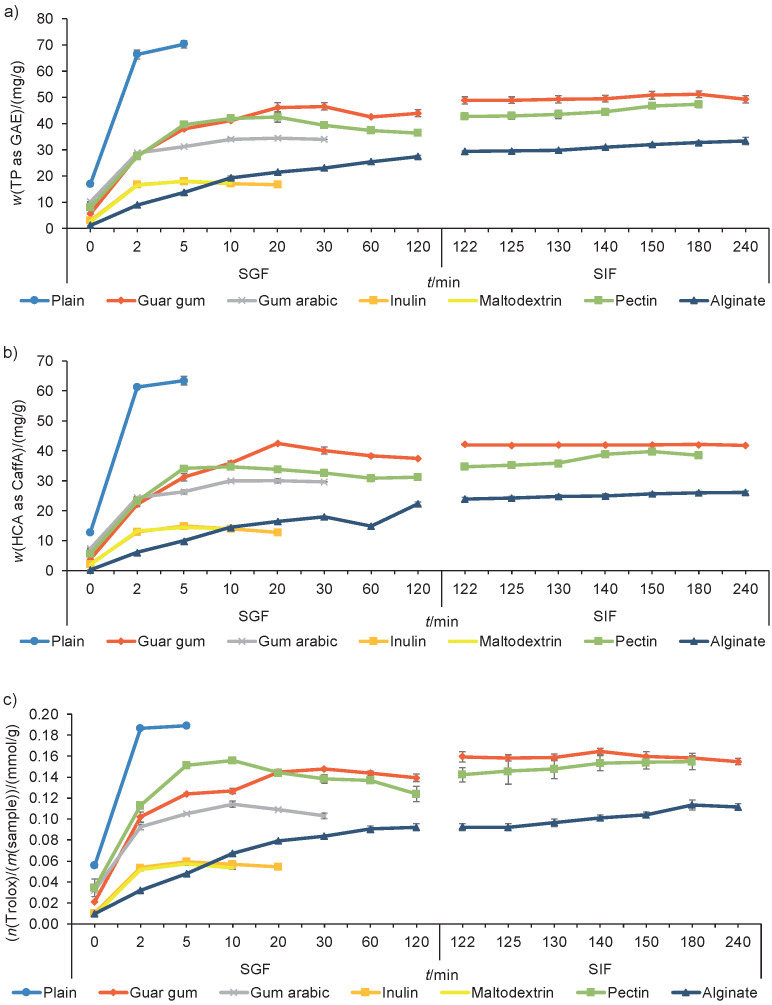
Release profiles of: a) total polyphenols (TP), b) hydroxycinnamic acids (HCA), and c) retained antioxidant capacity from plain dandelion powder and carrier-containing dandelion powders produced by spray drying in a simulated gastric (SGF) and intestinal (SIF) fluids. GAE=gallic acid equivalents, CaffA=caffeic acid

Although the highest content of the released TP (as gallic acid equivalents) was reported for plain dandelion powder (70.26 mg/g), this sample provided the fastest release of TP, where up to 100% was liberated in the first 5 min in SGF (Fig. 3a). On the other hand, an extended release rate of TP was reported for other carrier-containing dandelion powders, but in the end lower TP contents were released from these samples. Gum arabic-, inulin- and maltodextrin-dandelion powders released the TP completely in SGF, while guar gum-, pectin- and alginate-dandelion powders continued to release TP in SIF. Guar gum-dandelion powder showed a gradual release of TP during the first 30 min in SGF, whose release again continued in SIF, up to 180 min of analysis (51.20 mg/g). However, alginate-dandelion powder showed a gradual release of TP during all 240 min of analysis (33.38 mg/g).

A similar pattern was noticed in the release profiles of HCA and antioxidant capacity. After 5 min of analysis, plain dandelion powder released the fastest the HCA (*w*(CaffA)=63.45 mg/g) (Fig. 3b) and antioxidant capacity (*n*(Trolox)=0.189 mmol/g) (Fig. 3c). However, the released content of HCA and antioxidant capacity from plain dandelion powder was the highest compared to others. On the contrary, carrier-containing dandelion powder mostly enabled a longer gradual release of polyphenols and antioxidant capacity, but finally lower amount was discharged during analysis. Such reduced content of released polyphenols and antioxidant capacity in carrier-containing dandelion powders could be ascribed to the potential interactions between the dandelion polyphenols and polysaccharides from the applied carriers. When observing carrier-containing dandelion powders, the fastest release of both HCA and antioxidant capacity was reported for inulin- and maltodextrin-dandelion powders. They were totally degraded after 10 (maltodextrin-dandelion powder) and 20 min (inulin-dandelion powder) of analysis in SGF. However, they enabled a gradual release only for the first 5 min, after which the values decreased. Moreover, guar gum-, alginate- and pectin-dandelion powders mainly enabled prolonged release of HCA and antioxidant capacity in both SGF and SIF. Guar gum-dandelion powder enabled a gradual release of HCA for the first 20 min (*w*(CaffA)=42.51 mg/g) in SGF, while in SIF the content of HCA decreased or remained flat (*w*(CaffA)=41.84 mg/g, 240 min) (Fig 3b). Regarding release of antioxidant capacity, guar gum-dandelion powder allowed a gradual release for the first 30 min (*n*(Trolox)=0.148 mmol/g) in SGF. After transport in SIF, guar gum-dandelion powder again started releasing the antioxidant capacity until 140 min of analysis (*n*(Trolox)=0.164 mmol/g), after which the antioxidant capacity values decreased (Fig. 3c). However, this sample enabled the highest content of released HCA and antioxidant capacity among all carrier-containing dandelion powders. Although the content of released HCA and antioxidant capacity from alginate-dandelion powder was lower than of guar gum-dandelion powder, HCA content was gradually released from this sample during all 240 min of analysis (only exception in 60 min of SGF), or during 180 min for antioxidant capacity, which is preferable overall.

Moreover, it was noticed that the powders prepared with the highest carrier content (10% inulin and maltodextrin) enabled the fastest release of bioactive compounds at the end of analysis, accompanied by their lowest content. On the contrary, the sample containing the lowest carrier content (0.5% guar gum) released the highest content of bioactive compounds after 240 min of analysis. However, alginate-dandelion powder prepared with 4% of a carrier in the delivery solution was characterized as a sample with the longest gradual release of the evaluated compounds. Thus, if considering the released content and a favourable gradual release pattern, both the carrier type and its content affected the release profiles of polyphenols and antioxidant capacity from the examined dandelion powders.

## CONCLUSIONS

Dandelion (*Taraxacum officinale* L.) leaf extract has been for the first time microencapsulated successfully in different carrier materials using spray drying at low inlet temperature (130 °C). The results showed that the carrier type influenced more the wettability, bulk density, Carr index, Hausner ratio, particle size distribution width, morphological and encapsulation properties, while the content of the applied carriers had a higher effect on the moisture content, solubility and *d*(0.5) of the evaluated dandelion powders. However, both the carrier type and its content affected the colour characteristics, hydroxycinnamic acid (HCA) content and the release properties of the examined samples. Among the carrier-containing dandelion powders, guar gum-dandelion powder exhibited the highest solubility, the lowest total colour difference (a favourable trait) and the highest HCA content, but also the highest moisture content. Although inulin and maltodextrin are often used as carriers for spray drying of polyphenol-rich extracts, the obtained results implied that they were not suitable carriers for encapsulation of dandelion leaf extract (the lowest encapsulation efficiency and the fastest liberation of polyphenols and antioxidant capacity from dandelion powders). On the other hand, they had the most desirable morphological properties and the lowest *d*(0.5) and moisture content, which explains their wide usage in spray drying. Although pectin is not often used as a delivery vehicle for the encapsulation based on drying, here pectin-dandelion powder enabled the highest retention of polyphenols and antioxidant capacity. These results could open a new direction for examination of pectin in spray drying of plant extracts. Furthermore, alginate-dandelion powder provided preferably the longest gradual release of all evaluated compounds in simulated gastrointestinal conditions, which highlighted alginate-dandelion powder as a sample with the best release properties of dandelion polyphenols. Also, this sample was characterized by the most acceptable bulk density, flow and cohesive properties. Depending on a final purpose of powders, all employed carriers fulfil either physicochemical properties or encapsulation parameters, which in the end justifies their usage in this experiment. Results obtained from this study could be of high importance for scientific researchers in the field of preservation and microencapsulation of sensitive bioactive compounds. Since the produced dandelion powders are rich in polyphenols and are characterized as powders with good physicochemical characteristics, these results also have great impact on the functional food industry, where such powders could serve for the production and enrichment of various food products, especially instant powders. Although the study gave good initial results, further studies are required to determine the optimal carriers aimed for the production of dandelion powders with the best physicochemical properties (emphasis on enhancing flow properties) and even higher polyphenol loading capacities.
